# Renal-Limited Thrombotic Microangiopathy Presenting as Acute on Chronic Kidney Failure in the Absence of Schistocytosis

**DOI:** 10.7759/cureus.116417

**Published:** 2026-09-17

**Authors:** Raylee E Warren, Gloria Elizondo, Kyle Mitchell, Daniel Shay, Sufiyan Kamal, Bhavani Suryadevara, Angela Sutherland

**Affiliations:** 1 School of Medicine, The University of Texas Health Science Center at Tyler, Tyler, USA; 2 Internal Medicine, The University of Texas Health Science Center at Tyler, Tyler, USA; 3 Wound Care, The University of Texas Health Science Center at Tyler, Tyler, USA

**Keywords:** acute renal failure, dialysis, hypertension, stasis dermatitis, thrombotic microangiopathy

## Abstract

We report a rare presentation of renal-limited thrombotic microangiopathy (TMA). TMA is a clinicopathologic syndrome resulting from endothelial injury and activation of the coagulation and platelet pathways, leading to the formation of platelet-rich microthrombi within small vessels. This loss of blood flow due to the thrombi can result in tissue ischemia and end-organ injury. Systemic TMA classically manifests with the triad of microangiopathic hemolytic anemia (MAHA), thrombocytopenia, and organ dysfunction. However, TMA may be limited predominantly to the kidneys, resulting in acute or progressive renal dysfunction without the classic systemic hematologic manifestations, making diagnosis challenging. In this case, a 46-year-old woman with stage three chronic kidney disease (CKD) and untreated hypertension (HTN) presented with acute renal failure and chest pain. The initial differential diagnoses included systemic lupus erythematosus, glomerular disease, vasculitis, and TMA. An extensive diagnostic evaluation including peripheral blood smear, autoimmune serologies, and skin biopsy was largely unrevealing until kidney biopsy demonstrated findings consistent with TMA. She was ultimately diagnosed with renal-limited TMA and discharged with plans to initiate outpatient peritoneal dialysis for her newly progressed stage five CKD. This case highlights the diagnostic challenge of renal-limited TMA and emphasizes the importance of considering TMA in patients with otherwise unexplained acute or progressive renal dysfunction, even in the absence of the classic hematologic findings.

## Introduction

Thrombotic microangiopathy (TMA) is a rare disorder that is continuously being re-evaluated for naming, organization, and structure of subtypes, but is currently organized into hereditary and acquired disorders based on the underlying cause. Regardless of the specific subtype, TMA is generally characterized by endothelial injury, microvascular thrombosis, thrombocytopenia, microangiopathic hemolytic anemia (MAHA), and end-organ injury [1-3]. Common end-organs that are affected include the kidney and central nervous system (CNS). The most frequent histopathological findings seen in TMA with renal involvement include arteriolar thrombosis, intimal edema or thickening, and glomerular ischemic changes [4]. That being said, not all TMAs present with the classic symptoms stated above, and the clinical and histologic pictures can change based on acute vs chronic disease at the time of presentation [2,3].

We report a diagnostically challenging case of TMA for this exact reason. Given the clinical ambiguity on presentation, the differential diagnosis included thrombotic thrombocytopenic purpura (TTP), atypical hemolytic uremic syndrome (HUS), and lupus nephritis. In general, TMA presentations are physiologically and clinically complex and diagnostically challenging [3,5]. In a 2024 French cohort of patients with TMA, renal-limited TMA accounted for 45% of cases [5]. However, the prevalence of renal-limited TMA in the broader population remains uncertain given its rarity. More studies are needed to establish an overall prevalence for this condition.

The findings that made this case particularly challenging included the initial presentation of acute on chronic renal failure and chest pain, the lack of schistocytes on peripheral blood smear, normal ADAMTS13, and a broad autoimmune workup that was all negative. Additionally, the chronic lower extremity rash initially raised concern for a vasculitis etiology or connective tissue disease. All of these findings culminated in the necessity for kidney biopsy to confirm the diagnosis. This case highlights the importance of maintaining suspicion for renal-limited TMA even in the absence of classic findings [2,3,5].

## Case presentation

A 46-year-old woman presented with acute renal failure and chest pain. She was also found to have a chronic, recurrent skin rash on her right lower extremity (Figures 1-2). She had two days of throbbing, pressure-like chest pain with associated shortness of breath and sternal discomfort that awoke her from sleep. On arrival to the emergency department, she received sublingual nitroglycerin that relieved her chest discomfort. She denied any urinary symptoms, recent nonsteroidal anti-inflammatory drugs or antibiotics, and medication changes. She did endorse the aforementioned recurrent skin rashes that self-resolve without intervention. Of note, she reported four living children with three early term miscarriages and was currently menstruating regularly. She reported a 20-pound intentional weight loss with diet and exercise. She had a past medical history of hypertension but was not on any medications for it.

**Figure 1:**
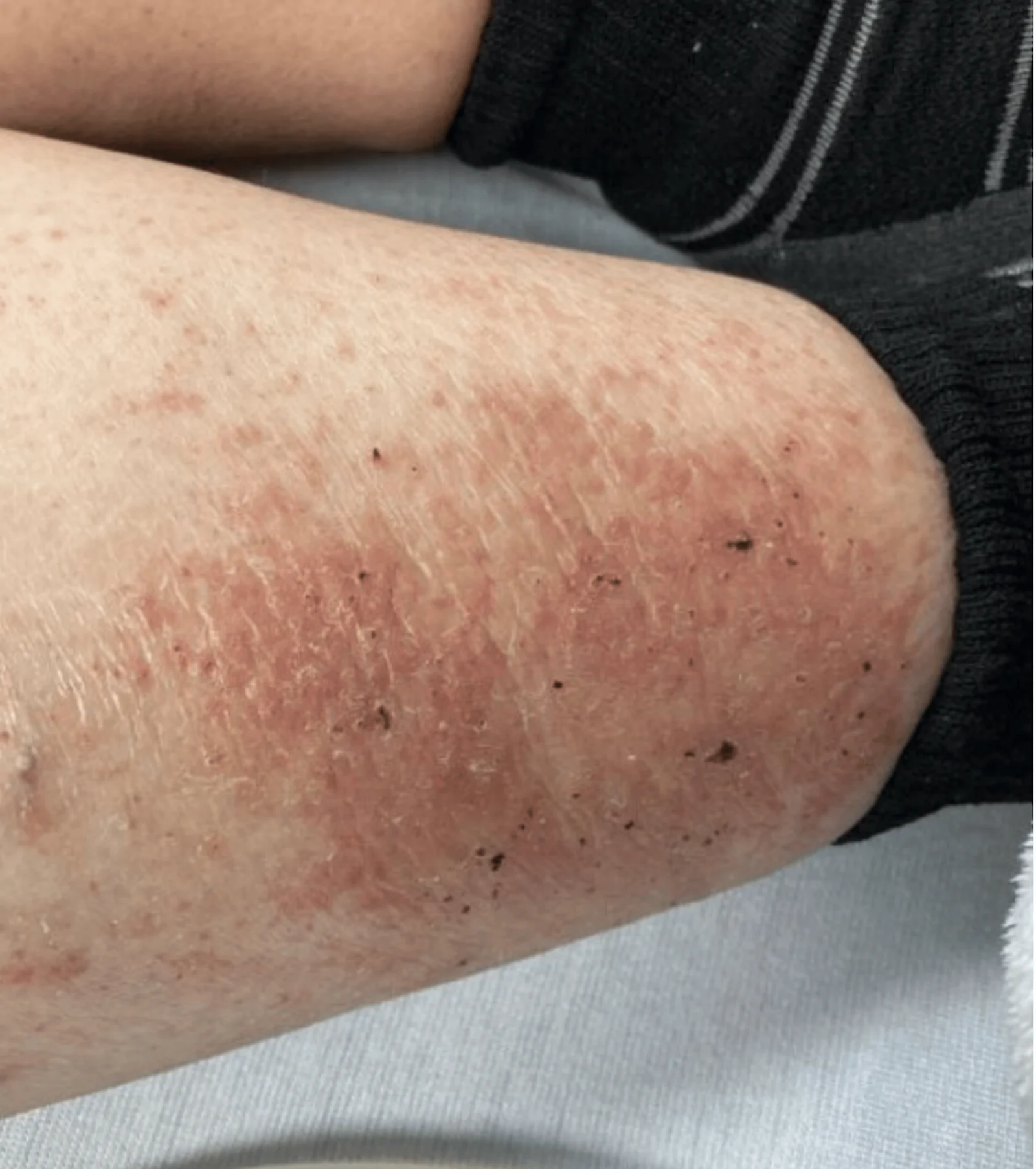
Right lower extremity rash, photo taken upon admission.

**Figure 2:**
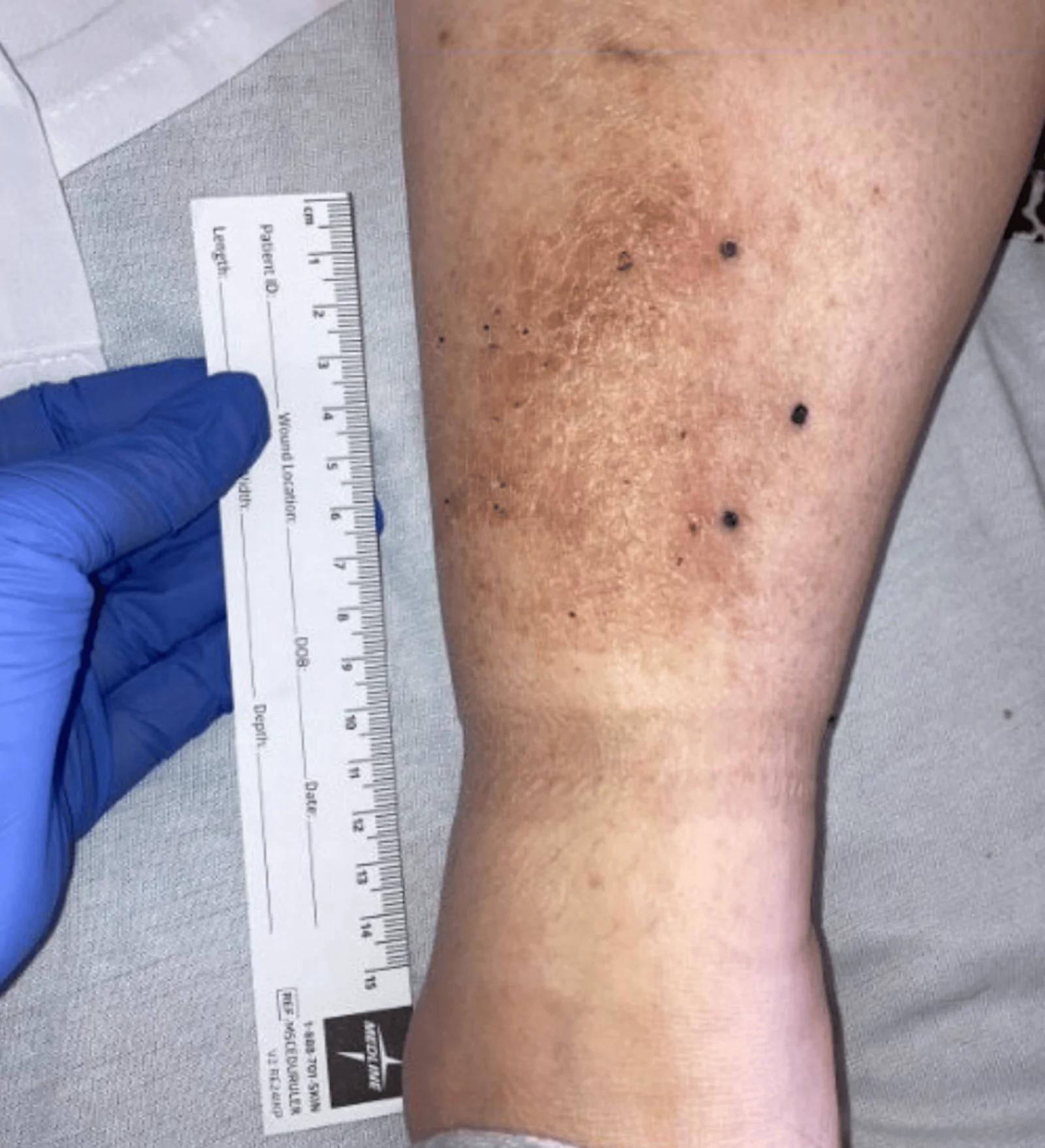
Right lower extremity rash approximately a week after initial photo and status post punch biopsies times three.

Vitals on admission were afebrile, blood pressure 148/87, and oxygen saturation 93% on 2.5L nasal cannula. Physical exam was positive for swelling of bilateral lower extremities, bilateral nonblanching petechiae, and right lower extremity erythematous rash up to the sock border. Initial labs are in Table 1. Her troponin had flat kinetics, which indicated renal insufficiency rather than NSTEMI proper. Workup primarily focused on the acute renal failure with a history of chronic kidney disease (CKD) stage three. Urine analysis was significant for protein of 366 mg/dL and a protein/creatinine ratio of 1.73. These findings warranted a renal ultrasound that demonstrated medical renal disease without hydronephrosis or cysts, and then a renal biopsy was obtained. Given the unusual presentation and preliminary findings, antiglomerular basement membrane (anti-GBM), antineutrophil cytoplasmic antibodies (ANCA), anti-dsDNA, and antinuclear antibodies (ANA) were obtained and were all negative. Peripheral blood smear showed anisopoikilocytosis, negative for schistocytosis.

**Table 1:** Lab values of interest with associated reference ranges appropriate for our patient’s demographics. Reference ranges sourced from [6,7].

Lab	Value	Reference range
Blood urea nitrogen	65 mg/dL	8–20 mg/dL
Creatinine	6.4 mg/dL (baseline 2.0 mg/dL)	Female: 0.50–1.10 mg/dL
Estimated glomerular filtration rate	8 mL/min	Average for ages 40-49: 99 mL/min
Complement C3	120 mg/dL	100–233 mg/dL
Complement C4	27 mg/dL	14–48 mg/dL
White blood count	5.7 × 10^3^ cells/mm^3^	4.5–11.0 × 10^3^ cells/mm^3^
Hemoglobin	7.0 g/dL	Female: 12–16 g/dL
Mean corpuscular volume	91 fL	80–98 fL
Platelets	89/μL	150,000–450,000/μL
Calcium	7.3 mg/dL	8.2–10.2 mg/dL
Phosphorous	6.1 mg/dL	3.0–4.5 mg/dL
Lactate dehydrogenase	298 U/L	80–225 U/L
Reticulocyte	5.78% of red cells	0.5%–1.5% of red cells
Haptoglobin	117 mg/dL	83–267 mg/dL
ADAMTS13	97%	>=61%

The patient also had punch biopsies of her right lower extremity rash. The skin biopsies were sent out to a dermatopathologist and confirmed stasis dermatitis with thick-walled vessels forming clusters in the papillary dermis accompanied by variable fibrosis and extravasation of red blood cells (Figure 3).

**Figure 3:**
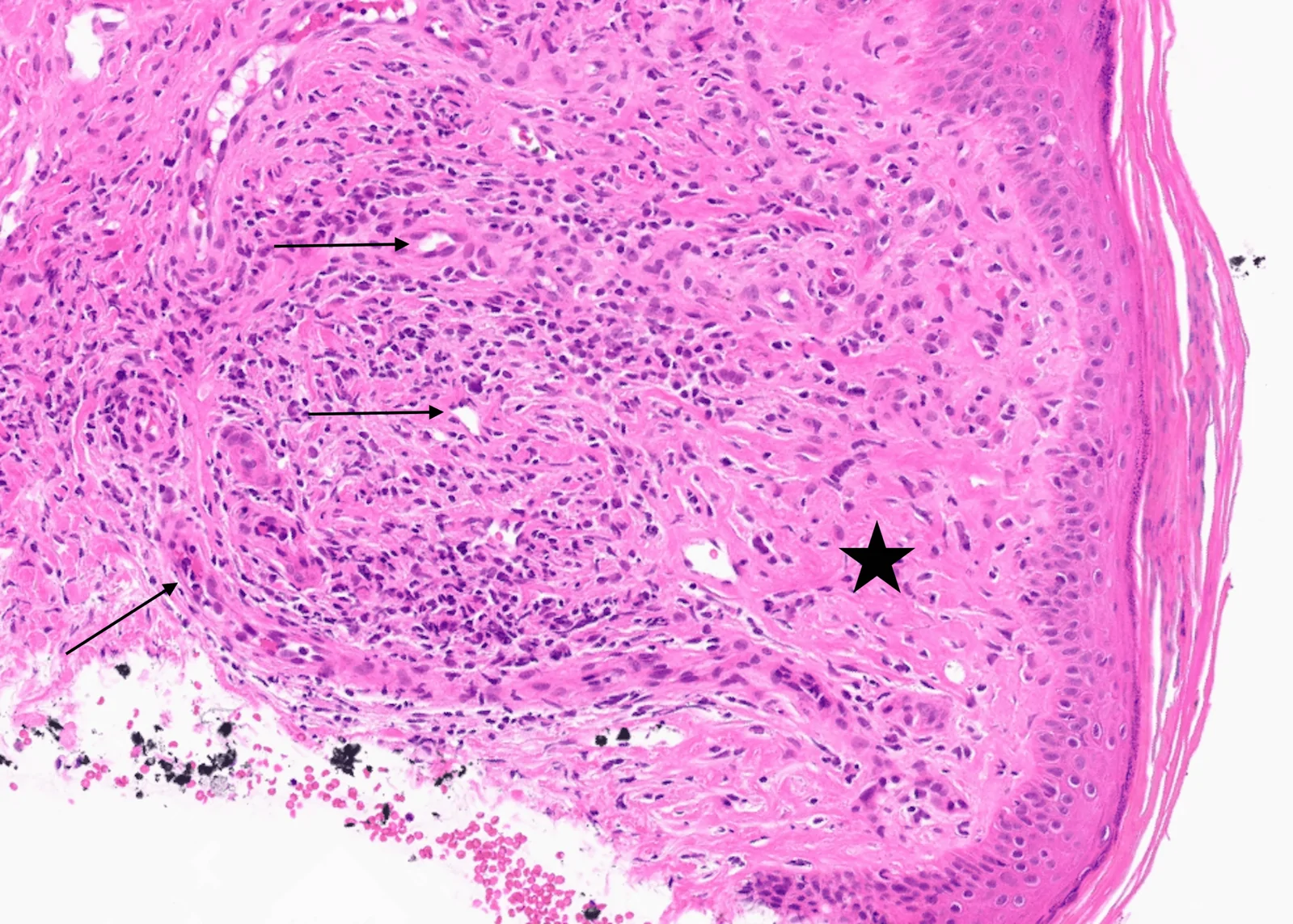
High-power light microscopy of right lower leg punch biopsy showing thick-walled vessels forming clusters in the papillary dermis accompanied by variable fibrosis. Black arrow: thick-walled vessels in papillary dermis Black star: area of fibrosis

Her kidney biopsy resulted in confirmed renal TMA and hypertension, now in CKD stage five. The biopsy showed no evidence of immune complex-mediated glomerulonephritis, but there was evidence of TMA mainly involving arterioles, which showed a combination of thrombosis and remodeling of the thickened intimal layer and focal segmental involvement of glomeruli (Figures 4-6). The slides were negative for all immunoreactants for immunofluorescence microscopy.

**Figure 4:**
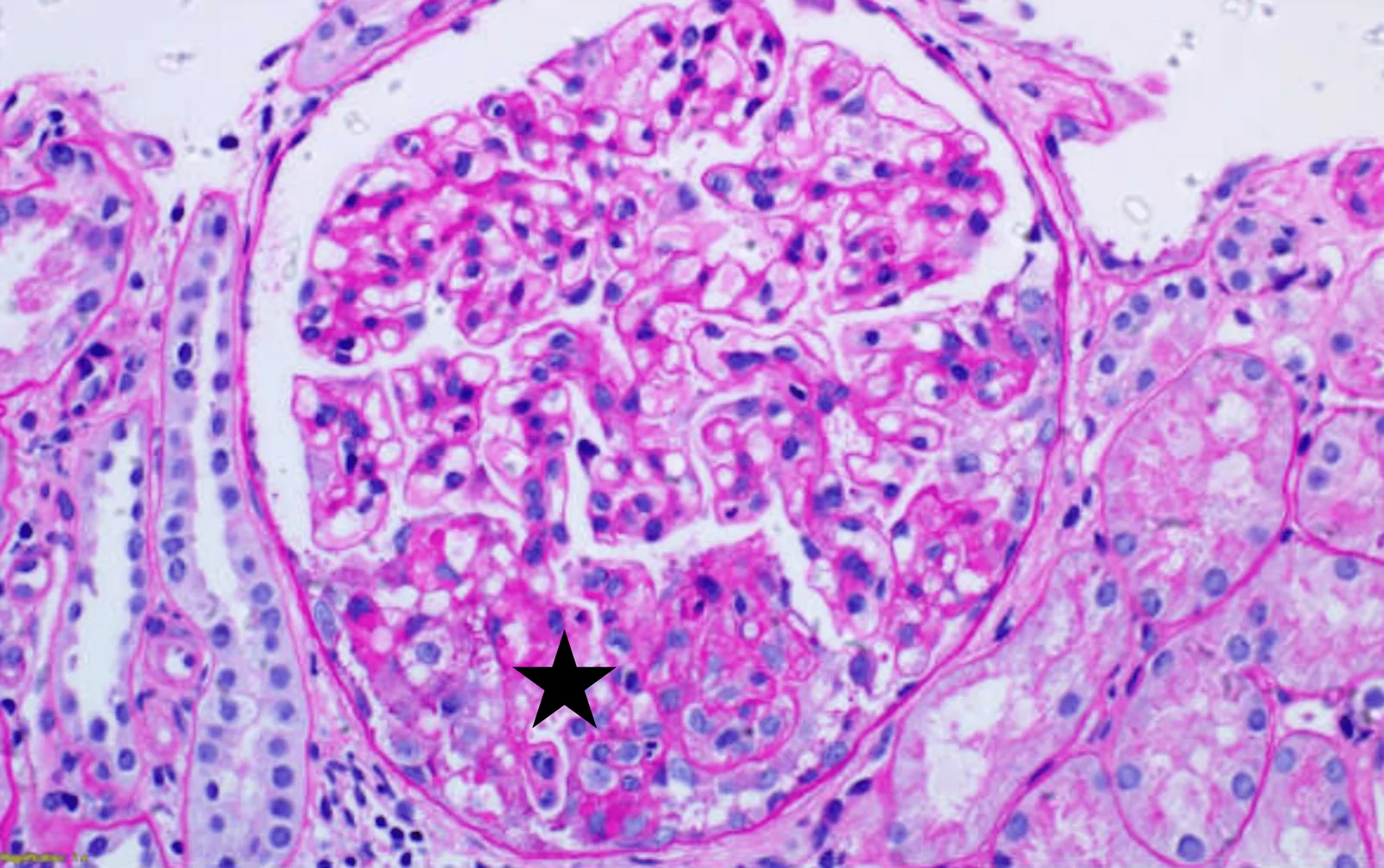
High-power light microscopy of kidney biopsy showing focal segmental proliferation of glomerulus. Black star: segmental proliferation within the glomerulus.

**Figure 5:**
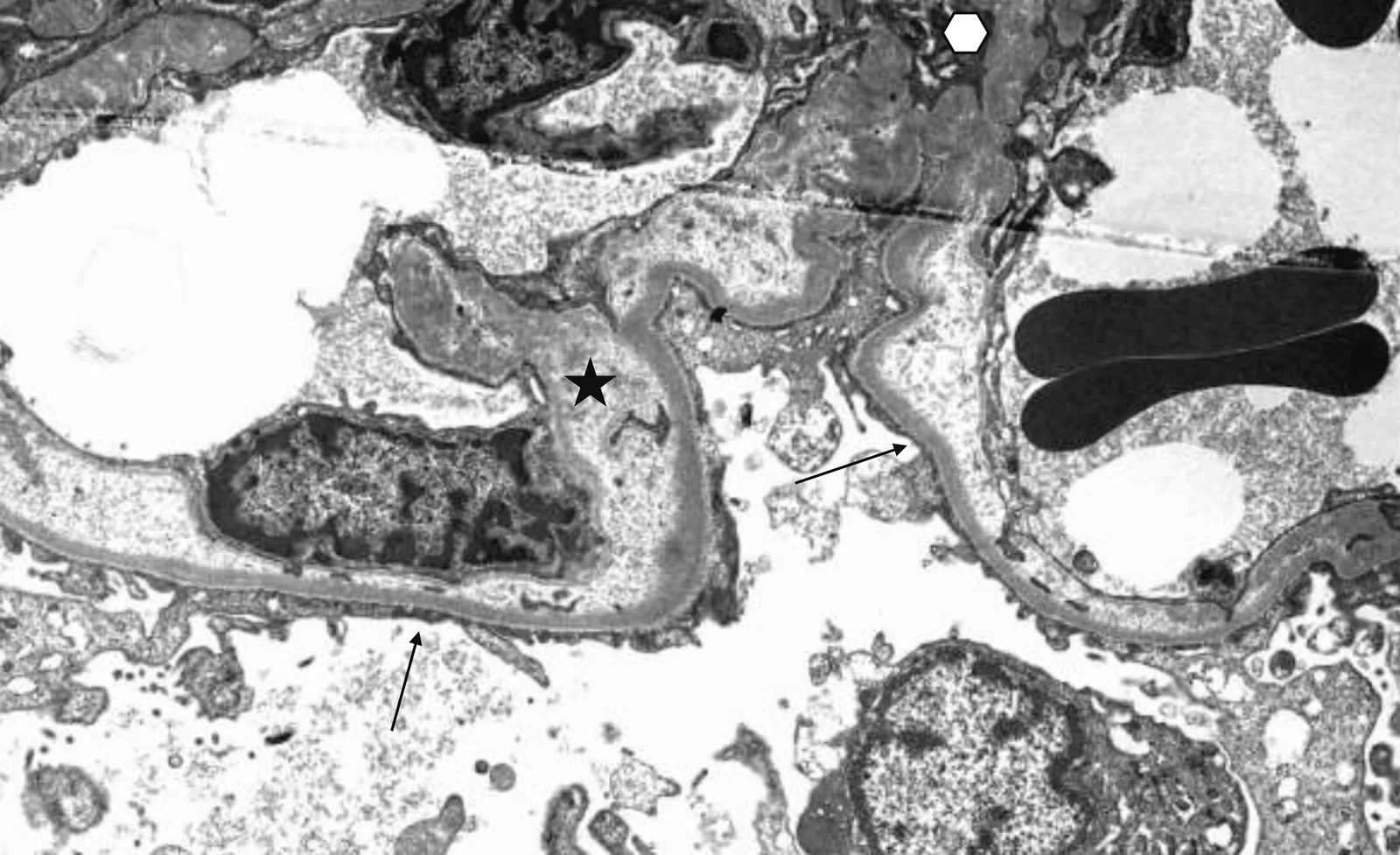
Electron microscopy of kidney biopsy showing normal glomerular basement membrane, swelling of endothelial cells, partial effacement of foot processes, and variable segmental ongoing lysis of matrix in the mesangial regions. Black arrows: effacement of podocyte foot processes with adjacent normal glomerular basement membrane Black star: swollen endothelial cell cytoplasm White hexagon: mesangial matrix lysis

**Figure 6:**
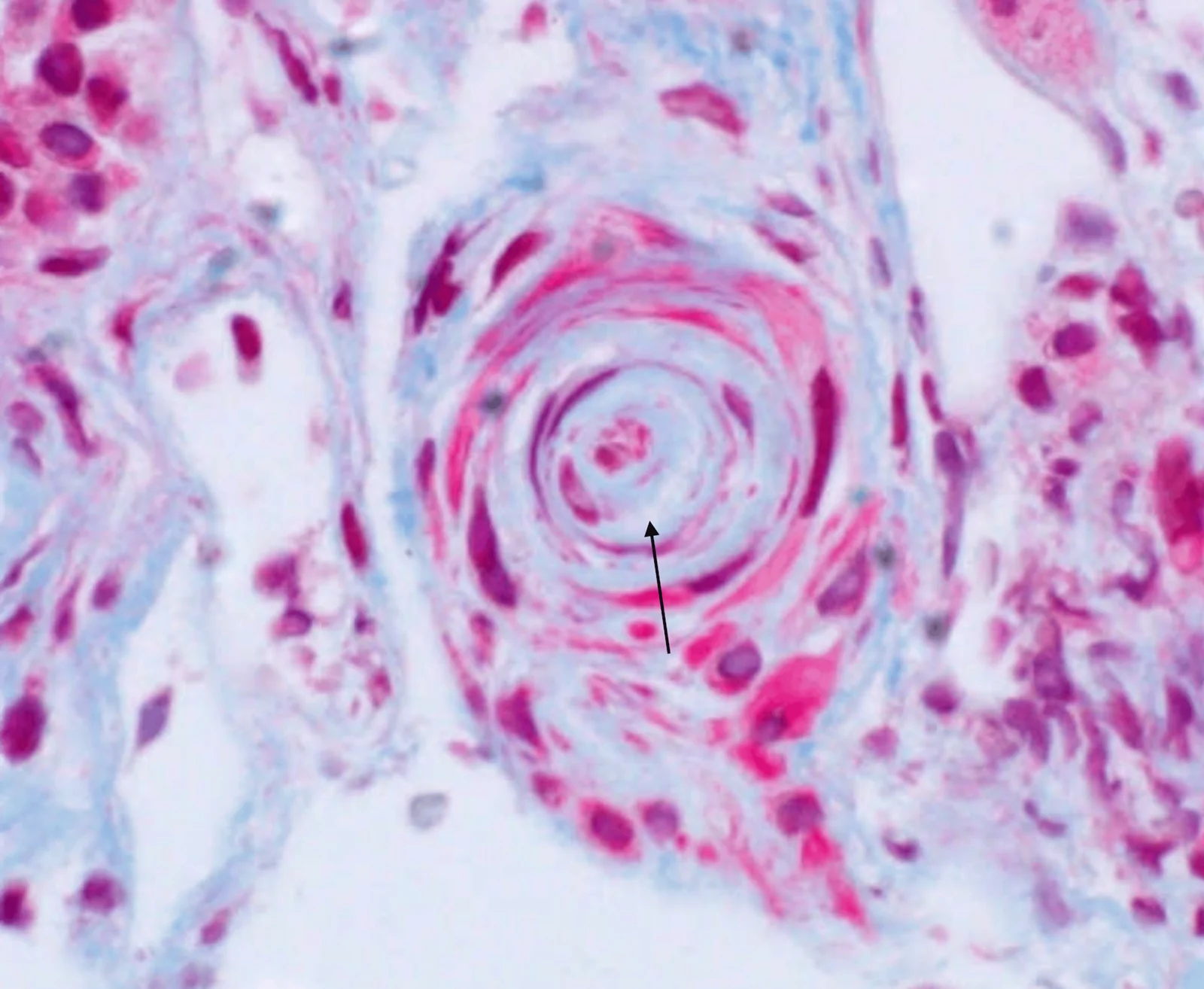
High-power light microscopy of kidney biopsy showing intimal concentric thickening. Black arrow: intimal concentric thickening

The patient was discharged on metoprolol tartrate 100 mg, nifedipine 60 mg, furosemide 80 mg, and hydralazine 50 mg, all twice daily. She was informed that dialysis would most likely be necessary, and upon follow-up with the nephrology outpatient department, the patient decided to pursue peritoneal dialysis at home. It is unknown whether the patient received outpatient genetic testing, antiphospholipid antibody testing, or other recommended laboratory testing to determine her TMA subtype.

## Discussion

TMA encompasses a heterogeneous group of disorders characterized by endothelial injury, microvascular thrombosis, thrombocytopenia, MAHA, and varying degrees of end-organ damage [1]. While the kidney is one of the most frequently affected organs, clinical recognition often relies upon the presence of overt hemolysis, schistocytosis, and thrombocytopenia, although these features may not always be present in chronic TMA [2,3]. This case illustrates the diagnostic difficulty posed by renal-predominant TMA, particularly when classical hematologic findings are absent and the presentation overlaps with more common causes of acute kidney injury.

Kidney biopsy was essential in establishing the diagnosis of TMA. Histopathologic findings included arteriolar thrombosis, intimal thickening and remodeling, focal glomerular involvement, and the absence of immune complex deposition. These findings are characteristic of chronic renal TMA but are not specific for a single underlying etiology [4]. Acute lesions typically demonstrate endothelial swelling and thrombus formation, whereas chronic lesions are characterized by concentric intimal fibrosis, vascular remodeling, and chronic ischemic glomerular collapse [4]. Because these morphological features overlap among multiple causes of TMA, renal biopsy confirms the presence of a thrombotic microangiopathic process but rarely identifies its precise cause in isolation. Clinicopathologic correlation remains essential for subtype classification [3].

The differential diagnosis initially included lupus nephritis, ANCA-associated vasculitis, anti-glomerular basement membrane disease, and TTP. Because complement activation, to varying degrees, is thought to be an underlying factor of TMA, many complement etiologies were considered [3]. Serologic testing was unrevealing, including negative ANA, anti-dsDNA, ANCA, and anti-GBM antibodies. Immunofluorescence microscopy demonstrated no evidence of immune-complex deposition, and skin biopsy revealed stasis dermatitis without vasculitis, making autoimmune and immune-complex mediated etiologies unlikely. The absence of immune deposits on biopsy further supported a vascular rather than inflammatory process. Because histologic features overlap considerably among TMA syndromes, exclusion of competing etiologies was necessary before attributing the biopsy findings to chronic renal-limited TMA [8]. 

A major diagnostic consideration was TTP. Severe ADAMTS13 deficiency, typically less than 10%, is highly sensitive for TTP and is typically accompanied by profound MAHA, marked thrombocytopenia, and only modest renal dysfunction [1]. In contrast, this patient demonstrated preserved ADAMTS13 activity with severe renal dysfunction and lacked convincing laboratory evidence of systemic hemolysis. The patient had no evidence of schistocytosis, no reduced haptoglobin, and only mildly elevated LDH. Recognition that renal-limited TMA may occur without overt hemolysis is important, as reliance on classic hematologic findings alone may delay diagnosis and treatment until irreversible vascular remodeling has occurred [2,3].

Another important consideration was the relationship between severe hypertension and TMA. Malignant hypertension may both precipitate endothelial injury resulting in TMA and develop secondary to primary TMA-related renal ischemia, making determination of causality challenging. Histological findings can help differentiate between these and other subtypes and are further explained in Table 2 [9,10,11]. There is also overlap with complement-mediated TMA, antiphospholipid syndrome, and TMA from malignant HTN with complement abnormalities [11,12]. Although complement levels were normal in this patient, this does not exclude complement-mediated TMA [3,12]. Likewise, antiphospholipid syndrome nephropathy remains an important diagnostic consideration because chronic vascular lesions may closely resemble other pathologies; therefore, antiphospholipid antibody testing should be performed when clinical suspicion exists [10]. The patient was encouraged to pursue this testing in the outpatient setting given her history of miscarriages, but it is unclear if this was completed. Collectively, these overlapping clinicopathologic features prevented confident assignment to a single TMA subtype and instead supported a diagnosis of chronic renal-limited TMA of uncertain etiology.

**Table 2:** Histologic features of overlapping TMA subtypes and clinical clues that can aid in the diagnosis. Information collected and combined into one cohesive table to visualize how to distinguish between common TMA subtypes [9-11].

Etiology	Histologic Features	Clinical Clues
Hypertension alone	Hyaline arteriolosclerosis, fibroelastic intimal thickening, global glomerulosclerosis	Longstanding HTN without thrombocytopenia or MAHA
Hypertension-associated TMA	Fibrinoid necrosis, endothelial swelling, platelet/fibrin thrombi, mesangiolysis	Severe or malignant HTN, AKI
Complement-mediated TMA (aHUS)	Glomerular capillary thrombi, endothelial swelling, mesangiolysis, double-contour GBMs, chronic vascular remodeling	May have normal complement levels, genetic abnormalities common
APS nephropathy	Fibrous intimal hyperplasia, organized/recanalized thrombi, focal cortical atrophy, fibrous arterial occlusion	Positive lupus anticoagulant, anticardiolipin, or beta-2-glycoprotein I antibodies

Despite advances in the understanding of complement-mediated disease and other TMA subtypes, delayed diagnosis remains associated with progression to end-stage kidney disease and dialysis dependence [3,9,13]. The long-term prognosis of renal TMA is influenced by the extent of irreversible injury such as glomerular ischemia, interstitial fibrosis, and chronic arteriolar injury [14,15]. Because of this patient’s chronic vascular remodeling and severe reduction in glomerular filtration rate, she progressed to CKD stage five and was discharged with plans for dialysis. The specific treatment and management of TMA is largely driven by the specific TMA subtype; therefore, since the subtype could not be established during the inpatient course, dialysis was the main treatment discussed prior to discharge [2,3]. Since discharge, the patient has decided to pursue peritoneal dialysis. This case illustrates that delayed recognition of renal-limited TMA may permit irreversible vascular damage before classic systemic manifestations become apparent.

## Conclusions

This case highlights that renal-limited TMA may occur without schistocytosis or overt microangiopathic hemolytic anemia and should remain a diagnostic consideration in patients with unexplained acute-on-chronic kidney injury, thrombocytopenia, and severe hypertension. Prognosis is largely determined by the extent of irreversible vascular and parenchymal injury at diagnosis, underscoring the importance of early recognition and kidney biopsy before chronic vascular remodeling, glomerular ischemia, and fibrosis develop. In patients with concurrent extrarenal findings, clinicopathologic correlation and targeted evaluation, including skin biopsy as in this case, can help exclude alternative autoimmune and immune complex-mediated processes. Ultimately, careful integration of clinical and histologic findings with multidisciplinary evaluation is essential for establishing the diagnosis of renal-limited TMA and identifying potentially reversible disease.
